# A multiplex PCR amplicon sequencing assay to screen genetic hearing loss variants in newborns

**DOI:** 10.1186/s12920-021-00906-1

**Published:** 2021-02-27

**Authors:** Haiyan Yang, Hongyu Luo, Guiwei Zhang, Junqing Zhang, Zhiyu Peng, Jiale Xiang

**Affiliations:** 1 BGI College, Zhengzhou University, Zhengzhou, 450001 China; 2 School of Life Sciences, Zhengzhou University, Zhengzhou, 450001 China; 3 BGI Education Center, University of Chinese Academy of Sciences, BGI Park, No.21 Hongan 3rd Street, Yantian District, Shenzhen, 518083 China; 4 BGI Genomics, BGI-Shenzhen, Shenzhen, 518083 China; 5 Tianjin Medical Laboratory, BGI-Tianjin, BGI-Shenzhen, Tianjin, 300308 China

**Keywords:** Hearing loss, Genetic screening, *GJB2*, *SLC26A4*, *MT-RNR1*

## Abstract

**Background:**

Congenital hearing loss is one of the most common birth defects. Early identification and management play a crucial role in improving patients’ communication and language acquisition. Previous studies demonstrated that genetic screening complements newborn hearing screening in clinical settings.

**Methods:**

We developed a multiplex PCR amplicon sequencing assay to sequence the full coding region of the *GJB2* gene, the most pathogenic variants of the *SLC26A4* gene, and hotspot variants in the *MT-RNR1* gene. The sensitivity, specificity, and reliability were validated via samples with known genotypes. Finally, a pilot study was performed on 300 anonymous dried blood samples.

**Results:**

Of 103 samples with known genotypes, the multiplex PCR amplicon sequencing assay accurately identified all the variants, demonstrating a 100% sensitivity and specificity. The consistency is high in the analysis of the test–retest reliability and internal consistency reliability. In the pilot study, 12.3% (37/300) of the newborns were found to carry at least one pathogenic variant, including 24, 10, and 3 from the *GJB2*, *SLC26A4*, and *MT-RNR1* gene, respectively. With an allele frequency of 2.2%, the NM_004004.6(*GJB2*):c.109G>A was the most prevalent variant in the study population.

**Conclusion:**

The multiplex PCR amplicon sequencing assay is an accurate and reliable test to detect hearing loss variants in the *GJB2*, *SLC26A4*, and *MT*-*RNR1* genes. It can be used to screen genetic hearing loss in newborns.

## Background

Congenital hearing loss is one of the most common birth defects in children. Around 30,000 newborns are estimated to born with congenital hearing loss every year in China [1]. Genetic factors and congenital cytomegalovirus infections accounted for approximately 60% and 21% of congenital hearing loss, respectively [2]. Early detection of congenital hearing loss is proven to be beneficial for language acquisition and academic performance [3].

Universal newborn hearing screening is performed in the nursery before discharge to discern the deaf and hard of hearing infants [4]. The methods include otoacoustic emission test and automated auditory brainstem response test [5]. Universal newborn hearing screening has been implemented in China for more than 20 years. It has made significant contributions to the early detection, diagnosis, and interventions of hearing loss [6], leading to improved language development [7]. However, it has limitations in identifying cytomegalovirus-induced hearing loss [8], aminoglycoside-antibiotic-induced ototoxicity [9], and delayed-onset prelingual hearing loss [10]. Additionally, the universal newborn hearing screening has a low positive predictive value, which may cause parental anxiety [11] and result in unnecessary follow-up tests [12]. These challenges urged the need for genetic screens to integrate into the universal newborn hearing screening program [13].

Genetic screens for hearing loss were initially proposed in 2006 [14]. Considering the highly genetic heterogeneity [15], a test covering hundreds of deafness-related genes was costly and hard to implement as a screening test in clinical settings. Limited genetic screens, targeting a limited number of variants, are feasible because hotspot variants existed and the ten most frequently encountered causative variants accounted for 30.4% of genetic diagnoses [5]. Large epidemiological studies demonstrated that *GJB2*, *SLC26A4*, and *MT-RNR1* are the most common disease-causing genes in the Chinese population [16, 17]. Recently, the clinical benefits of limited genetic screens of variants in these three genes were well studied [18–20].

Several techniques were developed to detect a limited number of variants. Wan et al. described a multiplex genetic screening system called the SNPscan assay technique to screen for 115 deafness-related variants [21]. Wang et al. developed a multicolor melting curve analysis-based assay to detect 12 deafness-related variants simultaneously [22]. Li et al. developed a PCR-reverse dot blot assay to screen 20 variants in hearing loss genes [23].

In this study, we developed a multiplex PCR amplicon sequencing assay to sequence the full coding region of the *GJB2* gene, the most pathogenic variants of the *SLC26A4* gene, and hotspot variants in the *MT-RNR1* gene. We then validated and piloted the genetic test in a newborn population.

## Material and methods

### Multiplex PCR

**Fig. 1:**
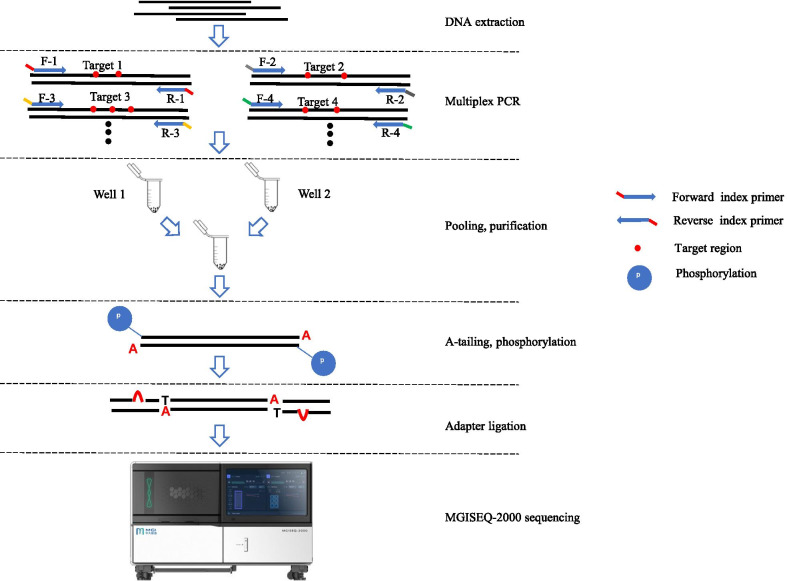
The workflow of the multiplex PCR amplicon sequencing assay

The workflow of the multiplex PCR amplicon sequencing assay is shown in Fig. 1. DNA was extracted from peripheral blood or dried blood spots by DNA Extraction Kit (BGI Biotech, Wuhan, China). Twenty-six pairs of indexed primers were used to amplify targeted regions by multiplex PCR (Additional file 1: Table S1). The amplification was conducted in a reaction mixture with a final volume of 25 µl containing 2 × KAPA 2G Fast Multiple PCR Mix (KAPA BIOSYSTEMS, Wilmington, MA, USA), 0.2uM Primer Mix, and 1–5 ng DNA templet. The PCR cycling program was as follows: 95 °C for 5 min; followed by ten cycles of denaturation at 95 °C for 30 s, annealing at 65 °C for 50 s (− 1 °C/cycle); then 25 cycles of denaturation at 95 °C for 30 s, annealing at 58 °C for 30 s; and a final extension at 72 °C for 15 s; 72 °C for the 60 s and holding at 12 °C.

### Library construction, sequencing, and analysis

PCR products of 48 samples (tag1-48) were pooled together. The pooled amplicons were purified and used for library construction. Briefly, 200 ng purified products were taken for end repair and A-tailing before adapter ligation. And then, the adapter-ligated-products were amplified by four-cycle PCR. Finally, after single-stranded circle formation and DNA nano balls preparation, the libraries were sequenced by MGISEQ-2000 sequencer (MGI, Shenzhen, China) in pair-end 100.

The cleaned reads were grouped to each sample based on index primers and then were mapped to the human reference genome (hg19) using Bowtie and SAMtools to create BAM and index files. Alignment data were next subjected to a strategic procedure for variant calling by GATK [24]. All hotspots are checked the mutation rate in bams and add the variants with a high mutation rate (> 0.1) and high depth (> 30X) as a complement to GATK detection. The genotype call is expressed as homozygous (allele fraction ≥ 0.8), heterozygous (0.1 ≤ allele fraction < 0.8), wildtype (allele fraction < 0.1).

### Validation and pilot of the assay

To validate the assay, we employed 103 dried blood samples with known genotypes to analyze the sensitivity and specificity, including 93 samples with positive genotypes and 11 samples with negative genotypes, respectively. Then, we performed experiments to access the test–retest reliability and internal consistency reliability. We used 14 samples and 31 samples with known genotypes in the analysis of the test–retest reliability and internal consistency reliability, respectively.

To test the performance of the assay, we randomly selected 300 anonymous dried blood samples. The detected genotypes were confirmed by sanger sequencing. This study was approved by the Institutional Review Board of BGI.

## Results

### Design and establishment of the assay

**Fig. 2:**
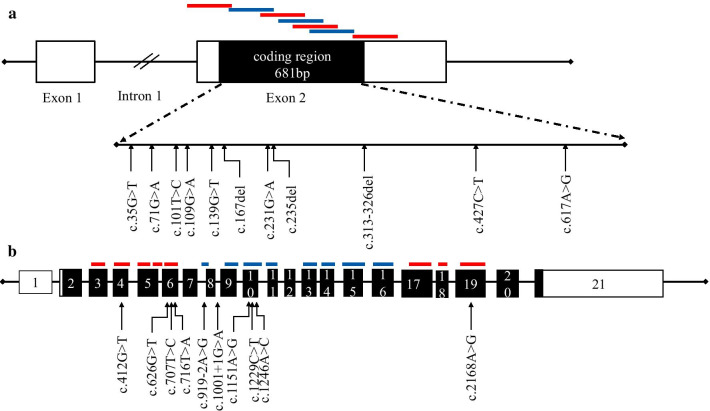
A schematic diagram showing the location of primers and hotspot variants. White boxes indicate exons, and the black boxes indicate the coding region. The lines above the coding regions indicate primers. The red lines present the primers in well 1, blue lines present the primers in well 2. The variants were pathogenic variants with an allele frequency greater than 1/10,000 in the gnomAD database

The workflow of the multiplex PCR amplicon sequencing assay is displayed in Fig. 1. To full sequence the coding region of the *GJB2* gene, the seven *GJB2* primers were divided into two groups for PCR reactions in parallel. The sixteen *SLC26A4* primers were divided to minimize the interaction between primers. As a result, well 1 included four primers in the *GJB2* gene, eight primers in the *SLC26A4* gene, three primers in the *MT-RNR1* gene; well 2 included three primers in the *GJB2* gene and eight primers in the *SLC26A4* gene (Fig. 2). The amplicons were then mixed into a single tube for library construction and referred to as sequencing.

Finally, the coding region of the *GJB2* gene was completely covered (Fig. 2a). Although only 63% of the coding region of the *SLC26A4* gene was covered, the known pathogenic variants in the ClinVar database with an allele frequency greater than 1/10,000 were all included (Fig. 2b). Additionally, three mitochondrial variants (m.1095T>C, m.1494C>T, and m.1555A>G) were included. The m.1494C>T and m.1555A>G are prevalent in 0.23% of the Chinese population [19]. The prevalence of m.1095T>C was 0.61% in a large cohort of 1642 Han Chinese pediatric subjects with aminoglycoside-induced and nonsyndromic hearing loss [25].

### Analytical studies

To verify the sensitivity and specificity of the multiplex PCR amplicon sequencing assay, we selected 11 negative and 92 positive samples with known genotypes (101 variants in total), including 65 heterozygous and 1 homozygous states in the *GJB2* gene; 30 heterozygous states in the *SLC26A4* gene; and 5 mitochondrial variants (4 homoplasmy and 1 heteroplasmy) in the *MT-RNR1* gene. The variants were all confirmed by Sanger sequencing. Our assay accurately identified all the 101 variants from the positive samples, indicating a 100% sensitivity (Table 1). No pathogenic variants were identified in the targeted region from negative samples, indicating a 100% specificity.

**Table 1:** The sensitivity of the multiplex PCR amplicon sequencing assay

Gene	Nucleotide variant	Homozygous	Heterozygous	Homoplasmy	Heteroplasmy	Total	Sensitivity (%)
*GJB2*	c.235delC	0	43	–	–	43	100
*SLC26A4*	c.919-2A>G	0	20	–	–	20	100
*GJB2*	c.109G>A	1	13	–	–	14	100
*SLC26A4*	c.1229C>T	0	9	–	–	9	100
*GJB2*	c.299_300delAT	0	9	–	–	9	100
*MT-RNR1*	m.1555A>G	–	–	4	1	5	100
*SLC26A4*	c.1707 + 5G>A	0	1	–	–	1	100
Total	–	1	95	4	1	101	100

**Fig. 3:**
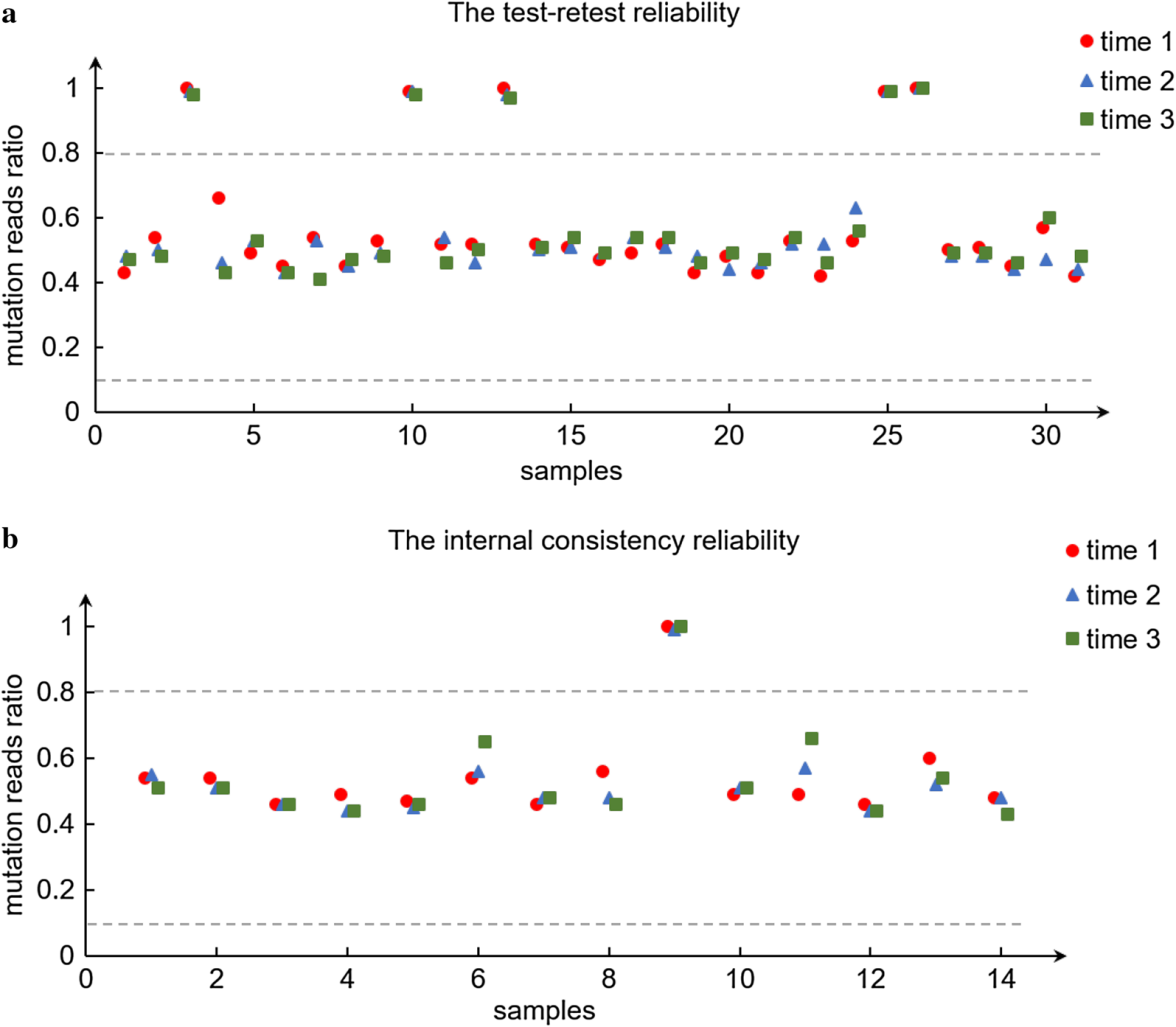
The reliability of the multiplex PCR amplicon sequencing assay. In the analysis of test–retest reliability (**a**), 31 samples with known genotypes were tested three independent times. In the analysis of internal consistency reliability (**b**), 14 samples with known genotypes were repeatedly tested in an internal test

To validate the assay, we analyzed the test–retest reliability and internal consistency reliability. In the test–retest reliability, we performed the assay on 31 samples with known genotypes three independent times. In the internal consistency reliability, 14 samples were repeatedly tested in an internal test. All the variants in a heterozygous state were identified in a ratio between 0.1 and 0.8 and the variants in a homozygous state were identified in a ratio greater than 0.8 (Fig. 3, Additional file 2: Table S2, Additional file 3: Table S3). There was no significant difference in the ratio for any of the samples. These results demonstrated the multiplex PCR amplicon sequencing assay has a high consistency in detecting variants in targeted regions.

### Pilot evaluation

Of 300 anonymous dried blood spots, 37 (12.3%) were found to carry at least one pathogenic variant. One sample was identified in a homozygous and three samples were identified in homoplasmy. All other samples were in a heterozygous state (Table 2). NM_004004.6:c.109G>A in the *GJB2* gene was the most prevalent variant in the cohort, accounting for an allele frequency of 2.2%. The complete sequence of the coding region in the *GJB2* gene revealed a carrier rate of 8% (24/300), contributed to c.109G>A (4.3%), c.235delC (2.7%), c.176_191del (0.7%), c.299_300delAT(0.3%). Additionally, four variants in the *SLC26A4* genes were identified; they are c.919-2A>G (1.3%), c.1975G>C (1.0%), c.589G>A (0.7%) and c.1173C>T (0.3%). The carrier rate of m.1095T>C in the *MT-RNR1* gene was 1.0%.

**Table 2:** Allele frequency and carrier rate of variants in a cohort of 300 newborns

Gene screening	Nucleotide variant	Homozygous	Heterozygous	Homoplasmy	Heteroplasmy	Total	Allele frequency	Carrier rate
*GJB2*	c.109G>A	0	13	–	–	13	0.022	0.043
*GJB2*	c.235delC	0	8	–	–	8	0.013	0.027
*SLC26A4*	c.919-2A>G	1	3	–	–	4	0.008	0.013
*SLC26A4*	c.1975G>C	0	3	–	–	3	0.005	0.010
*MT-RNR1*	m.1095T>C	–	–	3	–	3	–	0.010
*GJB2*	c.176_191del	0	2	–	–	2	0.003	0.007
*SLC26A4*	c.589G>A	0	2	–	–	2	0.003	0.007
*GJB2*	c.299_300delAT	0	1	–	–	1	0.002	0.003
*SLC26A4*	c.1173C>A	0	1	–	–	1	0.002	0.003
Total	–	1	33	3	0	37	–	0.123

## Discussion

Hearing loss is the most common neurosensory disorder in humans, with an incidence of one in 1000 worldwide [26]. The most common pathogenic variants were from three genes (*GJB2*, *SLC26A4*, and *MT-RNR1*) in the Chinese population [1, 27–29]. Previous studies primarily focused on a number of limited hotspot variants, such as NM_004004.6:c.235delC in the *GJB2* gene, NM_000441.2:c.919-2A>G in the *SLC26A4* gene, and m.1555A>G and m.1494C>T in the *MT-RNR1* gene [20]. In this study, we developed a multiplex PCR amplicon sequencing assay, covering the full coding region of the *GJB2* gene, the most pathogenic variants in the *SLC26A4* gene, and the three hotspot variants in the *MT-RNR1* gene.

In this study, the multiplex PCR amplicon sequencing assay was designed to cover the entire coding region of the *GJB2* gene. The implement of *GJB2* screening in newborns is considered necessary and feasible for the following reasons. First, *GJB2* is the most common gene causing congenital hearing loss [30]. It is estimated that the single gene contributed to 21% of the congenital hearing loss and 15% of the childhood hearing loss at 4 years [14]. Second, the coding region of the *GJB2* gene is short (681 bp) enough to achieve a complete sequence at a low cost. Third, previous studies mainly focused on several hotspot variants including NM_004004.6:c.235delC, NM_004004.6:c.299_ 300delAT, NM_004004.6:c.176del16, and NM_004004.6:c.35delG, et al. These studies identified some hearing loss patients with inconclusive genotypes (a heterozygous variant in a single gene) [19, 20]. The second variant might be identified by a complete sequence of the coding region in the *GJB2* gene.

Our study recovered pathogenic variants in the *GJB2* gene had a carrier rate (8%) in the newborn population. The high rate is attributable to the inclusion of NM_004004.6:c.109G>A (4.3%) in our assay. NM_004004.6:c.109G>A is the most prevalent variant in Eastern Asia [31]. Although the penetrance is low, it was proven to have strong associations with mild or moderate hearing loss [32]. Recently, the ClinGen Hearing Loss Expert Panel reached a consensus interpretation of this variant as a pathogenic variant [33]. The documentation of patients’ longitudinal auditory features with NM_004004.6(*GJB2*):c.109G>A supports including it in a screening panel [34].

The *SLC26A4* gene is another common cause of hearing loss. It is attributed to only 3% of the congenital hearing loss, but the proportion significantly increased to 12% at the age of four [14]. This is because that the pathogenic variants in the *SLC26A4* gene cause enlarged vestibular aqueduct, which is late-onset. More importantly, the late-onset hearing loss cannot be identified by conventional newborn hearing screens because the hearing status was normal at birth when the tests were performed. In this scenario, genetic screens are proven to be a complementary test to conventional newborn hearing screens [20]. In our study, the full coding region of the *SCL26A4* gene was not fully covered because it has over 20 coding regions. Still, we covered all the hotspot variants in the Chinese population (Fig. 2b), including c.919-2A>G, c.2168A>G, c.1975G>C, which accounts for 13.39% of the cases contributed to the *SLC26A4* gene in a cohort of 864 Chinese patients [35].

*MT-RNR1* is a mitochondrial gene associated with aminoglycoside-induced hearing loss [36]. Individuals carrying such variants have normal hearing functions until aminoglycoside exposure. The drug susceptibility is unable to be identified by conventional physiologic hearing screens but detectable by genetic screens [20]. This genetic information is not only useful for the newborns who received the genetic screens, but also beneficial for the maternal relatives because the mitochondrial variants are transmitted in maternal inheritance. In this study, 1% of newborns were identified to carry mitochondrial variants, higher than the proportion (0.24%) from the previous studies [18]. This might be attributable to the small sample size.

The genetic hearing loss test was proposed to incorporate into the newborn physiologic hearing screening program before hospital discharge [5]. The results of the genetic test are valuable for the choice of following-up tests. Considering a high risk of hearing loss, audiologic evaluations should be arranged directly if a homozygous or compound heterozygous status was identified [20]. This strategy can identify newborns with hearing loss missed by physiologic screens, whom may benefit from a prompt intervention and management [18–20]. If a newborn with a heterozygous variant failed the physiologic screens, a following-up physiologic re-test or a comprehensive genetic test should be suggested. Asymptomatic newborns with *MT-RNR1* variants and the maternal family members should be informed of their predisposition for aminoglycoside toxicity [37].

We employed a multiplex PCR amplicon sequencing assay to analyze variants in deaf-related genes for two reasons. First, multiplex PCR allows the simultaneous detection of multiple targets of interest in an easy and efficient way [38]. Second, this assay relied on the next-generation sequencing techniques, facilitating high throughput processing of a large number of samples in a short time. These characters allow the test to implement in a large population as a screening test.

## Conclusion

In conclusion, we developed and validated a multiplex PCR amplicon sequencing assay to detect variants in the *GJB2*, *SLC26A4*, and *MT-RNR1* genes. Our results demonstrated that the assay is an accurate and reliable test and can be used to screen genetic hearing loss in newborns.

## Supplementary Information

**Additional file 1: Table S1.** Primers of *GJB2*, *SLC24A4* and *MT-RNR1* gene.

**Additional file 2: Table S2.** The test–retest reliability on 31 samples with known genotypes. **Table S3**: The internal consistency reliability on 14 samples with known genotypes.

**Additional file 3: Table S3.** The internal consistency reliability on 14 samples with known genotypes.

## Data Availability

The data used and/or analyzed during the current study are available from the corresponding author on reasonable request. The data are not publicly available due to privacy or ethical restrictions.

## Abbreviations

PCR: Polymerase chain reaction
DNA: Deoxyribonucleic acid
