# Supplementary material for: A multiplex PCR amplicon sequencing assay to screen genetic hearing loss variants in newborns
Source: BMC Med Genomics. 2021 Feb 27;14:61. doi: 10.1186/s12920-021-00906-1 (PMC7913202; doi:10.1186/s12920-021-00906-1)
Supplement: Supplementary file 1 — Additional file 1: Table S1. Primers of GJB2, SLC24A4 and MT-RNR1 gene. [file 12920_2021_906_MOESM1_ESM.pdf]

Additional file 1: Table S1. Primers of *GJB2, SLC24A4 and MT-RNR1* gene

**Primer ID**	**Forward Primer (5'-3')**	**Reverse Primer (5'-3')**
GJB2-1-W1	GTCTCCCTGTTCTGTCCTAGC	TCCAATGCTGGTGGAGTGTT
GJB2-2-W2	CACGCTGCAGACGATCCTG	GCAGGGTGTTGCAGACAAAG
GJB2-3-W1	GGTGTGGGGAGATGAGCAG	CACGTGCATGGCCACTAGGA
GJB2-4-W2	CCACATCCGGCTATGGGC	TTCGATGCGGACCTTCTGG
GJB2-5-W1	GAGTGAATTTAAGGACATCGAGGAG	TGCATGGAGAAGCCGTCGTA
GJB2-6-W2	CGCCTTCATGTACGTCTTCTATGT	CAGCAGGATGCAAATTCCAGACA
GJB2-7-W1	GGCCCACGGAGAAGACTGT	TCCCTCTCATGCTGTCTATTTCTT
SLC26A4-8-W1	AAGAGAGCCTTTGGTGTGCT	ACCAGAACTCTCAATCTGCCAA
SLC26A4-9-W1	ATGTGCTTTCAGGGATGGCA	GCCAAAACACTTTAAACATGAGCA
SLC26A4-10-W1	TGCAGACACATTGAACATTTGTGAT	ATACAGTTCCATTGCTGCTGG
SLC26A4-11-W1	TACAGCTAGAGTCCTGATTGCCA	GGGGTCTTGCTTACTATTTTAGCAC
SLC26A4-12-W1	GGCTTGCAGATTGGATTCATAGTG	TTCTGGAATGAACAGTGACCCA
SLC26A4-13-W2	GAAAGTTCAGCATTATTTGGTTGAC	GGCTCCATATGAAATGGCAGTAG
SLC26A4-14-W2	TTGTTCTCGGAGATGCTGGC	ACCAGTTCAGCAAAAGGGCA
SLC26A4-15-W2	TCGTTGTCATCCAGTCTCTTCC	TGGATATCATAAGGCTGTTGTTCC
SLC26A4-16-W2	ACACAAGGGAGAAGGACGAATC	CAATCGGTATGCAGAGAAGCA
SLC26A4-17-W2	TTTTTCCCTAGGTTATCTGGGTGTT	TTGTTCTGTTTATAGGAAATCTGGG
SLC26A4-18-W2	ATTCCAAAATACGGCTGTTCCAA	CCAGCAAATGTCTCACAAAGGT
SLC26A4-19-W2	TGAAGAACCTCAAGGAGTGAAGATT	CTATTCCTGATTGGACCCCAGT
SLC26A4-20-W2	TAGGTGCCAGGCATTTTAAGTAAC	ACCTTTGTTGCTCTTAATTGTCCAC
SLC26A4-21-W1	AAGTGGATTGGAACTCTGAGC	ATTCAGAAAACCAGAACCTTACCAC
SLC26A4-22-W1	TATGGGCAGATAAGGTTGTTAATTG	GTGACCACAGTCCCAGATAGG
SLC26A4-23-W1	CAAAACATTGTGTCTTTCTTTTGA	TGGAACCTTGACCCTCTTGA
MT-RNR1-24-W1	CCAAGTCAATAGAAGCCGGC	TGGCGAGCAGTTTTGTTGAT
MT-RNR1-25-W1	GTACACACCGCCCGTCAC	AGGTTGTCTGGTAGTAAGGTGG
MT-RNR1-26-W1	GGACATTTAACTAAAACCCCTACGC	TTTGGCTAAGGTTGTCTGGTAGTAA
