# Supplementary material for: A multiplex PCR amplicon sequencing assay to screen genetic hearing loss variants in newborns
Source: BMC Med Genomics. 2021 Feb 27;14:61. doi: 10.1186/s12920-021-00906-1 (PMC7913202; doi:10.1186/s12920-021-00906-1)
Supplement: Supplementary file 2 — Additional file 2: Table S2. The test–retest reliability on 31 samples with known genotypes. Table S3: The internal consistency reliability on 14 samples with known genotypes. [file 12920_2021_906_MOESM2_ESM.pdf]

Additional file 2: Table S2. The test-retest reliability on 31 samples with known genotypes

Gene	Variant	Zygosity	Reads ratio		
			**time 1**	**time2**	**time3**
GJB2	c.109G>A	heterozygous	0.43	0.48	0.47
SLC26A4	c.919-2A>G	heterozygous	0.54	0.5	0.48
MT-RNR1	m.1095T>C	homoplasmy	1	0.99	0.98
GJB2	c.109G>A	heterozygous	0.66	0.46	0.43
GJB2	c.299_300delAT	heterozygous	0.49	0.53	0.53
GJB2	c.109G>A	heterozygous	0.45	0.43	0.43
SLC26A4	c.919-2A>G	heterozygous	0.54	0.53	0.41
GJB2	c.109G>A	heterozygous	0.45	0.45	0.47
GJB2	c.235delC	heterozygous	0.53	0.49	0.48
MT-RNR1	m.1095T>C	homoplasmy	0.99	0.99	0.98
SLC26A4	c.919-2A>G	heterozygous	0.52	0.54	0.46
GJB2	c.109G>A	heterozygous	0.52	0.46	0.5
MT-RNR1	m.1095T>C	homoplasmy	1	0.98	0.97
SLC26A4	1975G>C	heterozygous	0.52	0.5	0.51
GJB2	c.235delC	heterozygous	0.51	0.51	0.54
GJB2	c.109G>A	heterozygous	0.47	0.49	0.49
SLC26A4	c.919-2A>G	heterozygous	0.49	0.54	0.54
GJB2	c.235delC	heterozygous	0.52	0.51	0.54
GJB2	c.109G>A	heterozygous	0.43	0.48	0.46
GJB2	c.109G>A	heterozygous	0.48	0.44	0.49
GJB2	c.109G>A	heterozygous	0.43	0.46	0.47
GJB2	c.176_191del	heterozygous	0.53	0.52	0.54
GJB2	c.109G>A	heterozygous	0.42	0.52	0.46
GJB2	c.299_300delAT	heterozygous	0.53	0.63	0.56
MT-RNR1	m.1555A>G	homoplasmy	0.99	0.99	0.99
MT-RNR1	m.1095T>C	homoplasmy	1	1	1
GJB2	c.299_300delAT	heterozygous	0.5	0.48	0.49
GJB2	c.235delC	heterozygous	0.51	0.48	0.49
GJB2	c.109G>A	heterozygous	0.45	0.44	0.46
SLC26A4	c.919-2A>G	heterozygous	0.57	0.47	0.6
GJB2	c.109G>A	heterozygous	0.42	0.44	0.48
