# Supplementary material for: A multiplex PCR amplicon sequencing assay to screen genetic hearing loss variants in newborns
Source: BMC Med Genomics. 2021 Feb 27;14:61. doi: 10.1186/s12920-021-00906-1 (PMC7913202; doi:10.1186/s12920-021-00906-1)
Supplement: Supplementary file 3 — Additional file 3: Table S3. The internal consistency reliability on 14 samples with known genotypes. [file 12920_2021_906_MOESM3_ESM.pdf]

Additional file 3: Table S3. The internal consistency reliability on 14 samples with known genotypes

**Gene**	**Variant**	**Zygosity**	**Reads ratio**		
			**time 1**	**time 2**	**time 3**
SLC26A4	c.919-2A>G	heterozygous	0.54	0.55	0.51
GJB2	c.235delC	heterozygous	0.54	0.51	0.51
GJB2	c.109G>A	heterozygous	0.46	0.46	0.46
GJB2	c.109G>A	heterozygous	0.49	0.44	0.44
GJB2	c.109G>A	heterozygous	0.47	0.45	0.46
GJB2	c.176_191del	heterozygous	0.54	0.56	0.65
GJB2	c.109G>A	heterozygous	0.46	0.48	0.48
GJB2	c.299_300delAT	heterozygous	0.56	0.48	0.46
MT-RNR1	m.1095T>C	homoplasmy	1	0.99	1
GJB2	c.299_300delAT	heterozygous	0.49	0.51	0.51
GJB2	c.235delC	heterozygous	0.49	0.57	0.66
GJB2	c.109G>A	heterozygous	0.46	0.44	0.44
SLC26A4	c.919-2A>G	heterozygous	0.6	0.52	0.54
GJB2	c.109G>A	heterozygous	0.48	0.48	0.43
